# Influence of Proteolysis on the Binding Capacity of Flavor Compounds to Myofibrillar Proteins

**DOI:** 10.3390/foods11060891

**Published:** 2022-03-21

**Authors:** Hao Li, Rui Zheng, Fangfang Zuo, Chengyu Qian, Zhengan Yao, Ruipeng Dong, Di Zhao, Chunbao Li

**Affiliations:** College of Food Science and Technology, Nanjing Agricultural University, Nanjing 210095, China; 2019108026@njau.edu.cn (H.L.); zrui3825@163.com (R.Z.); zff9426986@163.com (F.Z.); 9191810625@njau.edu.cn (C.Q.); 9191810623@njau.edu.cn (Z.Y.); 9191810627@njau.edu.cn (R.D.)

**Keywords:** enzymatic hydrolysis, myofibrillar proteins, flavor compounds

## Abstract

Proteolysis occurs extensively during postmortem aging, enzymatic tenderization and fermentation of meat products, whereas less is understood regarding how proteolysis affects meat flavor. Myofibrillar proteins (MP) were extracted from beef longissimus dorsi muscle and subsequently treated with three commercial proteases. The effect of proteolysis on the interactions between the treated MP and butyraldehyde, 2-pentanone, octanal and 2-octanone was investigated. The progress of proteolysis increased the degree of hydrolysis (DH) and the surface hydrophobicity but decreased the turbidity and particle size. Fluorescence-quenching analysis results indicated that the enzymatic treatment generally increased the quenching constant (K_sv_) between the treated MP and ketones but decreased the K_sv_ between the treated MP and aldehydes, and the papain treatment changed the K_sv_ value to a larger degree than treatment with proteinase K and bromelain. The adsorption assay showed that the proteinase K treatment largely increased the adsorption capacity of the MP to octanal (by 15.8–19.3%), whereas the bromelain treatment significantly reduced the adsorption capacity of the treated MP to butyraldehyde (by 6.0–7.9%) and 2-pentanone (by 9.7–11.9%). A correlation analysis demonstrated a strong positive correlation (0.859, *p* < 0.05) between the DH of the MP and the adsorption ability of the treated MP to octanal. This study highlighted the significant but complex influence of proteolysis on MP binding capacity to flavor compounds.

## 1. Introduction

Meat and meat products constitute important sources of protein in a daily diet. Flavor is a key factor that determines the market potential and consumer satisfaction of meat products [1]. Raw meat is weakly-flavored but contains rich sources of compounds that are precursors of volatile compounds. Lipids, amino acids, fatty acids, peptides, reducing sugars and glycogenolysis products and glycolysis products are important contributors to meat flavor, through lipolysis oxidation and the Maillard reaction [1]. In addition, the thermal degradation of thiamine leads to the formation of thiazoles, thiophenes and furans [2]. Thousands of volatile compounds such as hydrocarbons, alcohols, aldehydes, ketones, carboxylic acids, esters, lactones, furans, pyrrans, pyrroles, pyrazines, pyridines, phenols, thiophenes, thiazoles, thiazolines, oxazoles, and other nitrogen and sulfuric compounds, are generated during thermal processing [1,3]. Comparatively, the flavors of meat products are also largely determined by the adsorption ability of meat components, such as water, proteins and lipids to flavor compounds. Proteolysis occurs extensively during the production and storage of meat. The hydrolysis of meat protein has been reported to occur during postmortem aging, enzymatic tenderization and the fermentation of meat products [4,5]. Both positive and negative relationships between proteolysis and the release of flavors in meat products were reported in several studies [5,6,7,8]. Excessive proteolysis in dry-cured ham liberated higher levels of free amino acids and increased the generation of hexanal, 2-methyl-butanoic acid and 3-methyl-butanoic acid through Strecker degradation [4]. Proteolysis of protein generates peptides and free amino acids, which were also reported to combine with volatile compounds, such as octanal, methional, 3-methylbutanal 2-pentanone, hexanal and 2-methylbutanal [9,10,11]. Whether the generated peptides and free amino acids have different abilities than intact proteins in adsorbing volatile compounds during proteolysis remains unclear. Our recent study indicated that mild enzymatic treatment by Flavourzyme and bromelain significantly elevated the levels of ketones and odors, whereas excessive proteolysis by papain and proteinase K largely reduced the levels of esters and aldehydes in beef longissimus dorsi [5]. Therefore, it was speculated that mild hydrolysis of proteins may expose more hydrophobic residues and increase the adsorption ability of meat proteins to flavor compounds, whereas the excessive proteolysis of protein may collapse the advanced structure of protein and reduce the interaction between meat protein and flavor compounds.

To verify these hypotheses, the influence of the enzymatic hydrolysis of myofibrillar proteins (MP) on the interaction between treated protein and flavor compounds, including 2-pentanone, 2-octanone, butyraldehyde and octanal was studied in this work. This research may assist in explaining the changes induced by proteolysis in the flavor of meat products, which may provide guidance for controlling the proteolysis of proteins during the processing and storage of meat and meat products.

## 2. Materials and Methods

### 2.1. Materials

Beef longissimus dorsi samples were bought from a local supermarket (Huarun Suguo Supermarket. Co., Ltd., Nanjing, China). The enzymes proteinase K (Pro K, 400 unit/mg), papain (Pap, 400 unit/mg) and bromelain (Bro, 500 unit/mg) were purchased from Solarbio Company (Beijing, China). Fluorescamine and leucine were purchased from Sigma-Aldrich (Shanghai, China). All the standard flavor substances, including 2-pentanone (>99.0%), 2-octanone (>99.5%), butyraldehyde (>99.5%), octanal (>99%) and methanol (>99.9%), were bought from Aladdin (Shanghai, China). The protease inhibitor Np-Tosyl-L-phenylalanine chloromethyl (TPCK) was obtained from Macklin (Shanghai, China).

### 2.2. Extraction of (MP) and Enzymatic Treatment

MP from the longissimus dorsi muscle of beef were extracted using the reported method in [12]. The biuret method [13] was conducted to determine the protein concentration using a BCA protein assay kit (Thermo Fisher, Waltham, MA, USA). The extracted protein was dissolved in phosphate-buffered saline (PBS, 20 mmol/L, pH 6.25) to a final concentration of 10 mg/mL. Pap, Pro K, and Bro prepared in the same buffer were added (0.25%, *w*/*w*) and then incubated at 30 °C using a water bath for 0, 5, 15, 30, and 60 min. Samples (2 mL) were collected and immediately reacted with 10 μL of protease inhibitor, TPCK (1 mg/mL, dissolved in methanol), to terminate the reaction. The control sample was prepared in the same way without the addition of an enzyme. Each sample was prepared in triplicate.

### 2.3. Degree of Hydrolysis (DH)

The degree of protease treatment was measured using a fluorescamine method [14]. A total of 30 μL of the treated MP sample was mixed with 900 μL of sodium tetraborate buffer solution (0.1 mol/L, pH = 8.0), and 300 μL of fluorescamine (0.2 mg/mL, diluted in acetone) solution was subsequently added. Afterwards, the mixture was kept in the dark for 30 min before measurement. The fluorescence characteristics were tested at the excitation and emission wavelengths of 390 nm and 480 nm, respectively, using a SpectraMax M2e microplate reader (Molecular Devices, Sunnyvale, CA, USA). Leucine (0, 0.5, 0.75, 1, 1.5, 2 and 3 mM) was used as standard to calculate the liberated free primary amino group (−NH_2_). The DH was calculated by the following equation:

$$\mathrm{DH}=\frac{\left[-{\mathrm{NH}}_{2}\left(h\right)\right]-\left[-{\mathrm{NH}}_{2}\left(0\right)\right]}{\left[-{\mathrm{NH}}_{2}\left(\infty \right)\right]-\left[-{\mathrm{NH}}_{2}\left(0\right)\right]}$$

[−NH_2_ (0)], [−NH_2_ (h)] and [−NH_2_ (∞)] represent the concentration of the free primary amino group when the MP were untreated, treated by protease, or completely hydrolyzed (6 M hydrochloric acid, 105 °C, 12 h), respectively.

### 2.4. Analysis of Sodium Dodecyl Sulfate-Polyacrylamide Gel Electrophoresis (SDS-PAGE)

MP (10 mg/mL) treated with enzymes at 0, 5, 15, 30, and 60 min were firstly diluted to 2 mg/mL and then fully mixed with the 4-fold loading buffer (GenScript, Piscataway, NJ, USA) at the ratio of 3:1 (*v*/*v*). Whereafter, each sample was immediately heated at 95 °C using a water bath for 5 min. After cooling, 10 μL of the samples or marker (5–270 kDa) was loaded into the 4–20% precast gels (GenScript, Piscataway, NJ, USA); then, the gels were run at 80 V for 30 min and at 120 V for another 1.5 h. Subsequently, the gels were stained with Coomassie blue G-250 for 30 min and destained with a solution composed of 250 mL of methanol and 75 mL of acetic acid. The gel images were acquired using an image scanner (GE Healthcare, Little Chalfont, Uppsala, Sweden).

### 2.5. Surface Hydrophobicity

The surface hydrophobicity of each sample was determined using a 1-anilino-8-naphthalene-sulfonate (ANS) method [15,16]. The protein samples, with or without protease treatment, were diluted to 1 mg/mL using PBS buffer (0.1 M, pH 7.0). Then, 20 μL of ANS (15 mM, 0.1 M PBS, pH 7.0) was mixed with 4 mL of the diluted protein solution and incubated in the dark for 20 min. The fluorescence of the mixed solution was measured using an SpectraMax M2e microplate reader (Molecular Devices, Sunnyvale, CA, USA) at the excitation wavelength of 375 nm and the emission wavelength of 470 nm.

### 2.6. Circular Dichroism Spectroscopy

The variation in the secondary structure of MPs, induced by enzymatic treatment, was analyzed by circular dichroism spectroscopy (Applied Photophysics, Surrey, UK). The MP (0.1 mg/mL) were loaded with a 1 cm quartz cell and the CD (circular dichroism) spectra were recorded in the wavelength range of 200–250 nm, at a scan rate of 50 nm/min, with 3 scans averaged for each CD spectra, corrected by the solvent signal.

### 2.7. Salt Solubility, Turbidity and Particle Size

For salt solubility, concentrations in the supernatant after centrifugation (5000× *g*, 20 min, 25 °C) of the MP suspension (10 mg/mL) were measured to calculate the ratio of salt-soluble fractions [17].

As for turbidity, after being diluted 10 times, the absorbance of the MP suspension (2 mg/mL) at 600 nm was recorded. Hydrazine sulfate solution (5 mL, 10 mg/mL) and 5 mL of hexamethylenetetramine solution (100 mg/mL) were mixed and diluted to 100 mL, and this suspension was defined as 400 Formazin turbidity units (FTU) according to Feng et al. [18].

The particle size was tested using a Zetasizer Nano ZS 90 (Malvern Instruments Ltd., Great Malvern, UK). The diluted MP suspension (0.1 mg/mL, 1.4 mL) was transferred to a quartz cuvette with a 1 cm path length, and the relative refractive index and absorption were set as 1.414 and 0.001, respectively [19]. Each sample was tested three times and the average particle size was calculated.

### 2.8. Fluorescence Spectra to Detect the Interaction between MP and Flavor Compounds

The reaction system consisted of 500 μL of the MP solution (3 mg/mL) and 4.3 mL of phosphate-buffered saline buffer, and 200 μL of the flavor compounds (0, 0.153, 0.307, 0.46, 0.613, 0.767, 0.92, 1.073 and 1.226 μL/mL, dissolved in methanol) were added later. The mixtures were fully mixed in headspace vials and then incubated at 20 ± 1 °C for 30 min. The spectra of the assay solutions were recorded using a fluorescence spectrophotometer (F-7000, Hitachi, Northeastern Honshu, Japan), equipped with 1 cm quartz cuvettes. The slit width was set as 5 nm and the scan speed was set as 1200 nm/min. The excitation wavelength was set at 282 nm, and the emission spectrum was recorded from 300 to 450 nm [20].

$$\frac{F_{0}}{F}=1+K_{\mathrm{SV}}\left[Q\right]$$

The Stern–Volmer quenching constant (K_sv_) of each compound was assessed by a linear regression plot of F_0_/F against [Q], where F_0_ and F represent the fluorescence intensities in the absence and presence of quencher, respectively, and [Q] is the quencher concentration.

The fluorescence-quenching rate constant (K_q_) was calculated according to the following formula, where τ_0_ refers to the fluorescence lifetime of biological macromolecules without the addition of quencher and equals approximately 10^−8^ s [21].

$$K_{q}=K_{\mathrm{SV}}/\tau_{0}$$

### 2.9. Adsorption Assay Evaluated by Headspace Gas Chromatography–Ion Mobility Spectrometry

The reaction system contained 4 mg/mL of the untreated/treated MP and 0.1 µL/mL of the flavors (diluted in methanol). After being incubated at 4 °C for 16 h in headspace vials, 500 µL of the headspace gas from the headspace vials was injected with a heated syringe needle (65 °C). To explore the changes in the adsorption capacity of the treated MP to flavor compounds, a GC-IMS (Gas Chromatography–Ion Mobility Spectrometry) instrument (FlavourSpec, G.A.S., Dortmund, Germany), equipped with an FS-SE-54-CB-1 (15 m × 0.53 mm × 1 μL) column, was used to determine the quantity of the free flavors in the headspace of the vials. The flavors were separated through the chromatographic column at 60 °C for 20 min, while nitrogen (99.99% purity) was used as the carrier gas. The programmed flow was set according to Hou et al. [22]. The flavors were identified by comparing the retention index (RI) and the drift time (DT) with the GC-IMS library, and the peak volume was recorded to calculate the percentages of the free flavor compound, according to the following formula:

$$\mathrm{free}\;\mathrm{flavor}\;\mathrm{compound}\;\left(\%\right)=\frac{{\mathrm{Peak}\;\mathrm{volume}}_{\mathrm{with}\;\mathrm{protein}\;\mathrm{added}}}{{\mathrm{Peak}\;\mathrm{volume}}_{\mathrm{without}\;\mathrm{protein}\;\mathrm{added}}}$$

where the free flavor compound (%) represents the flavor compounds in the headspace that were not adsorbed by proteins. The peak volume_with protein added_ represents the peak volume of the flavor compounds in the headspace of the vial that contained protein solution. The peak volume_without protein added_ represents the peak volume of the flavor compounds in the headspace of the blank control vial (containing only phosphate buffer, 20 mmol/L, pH 6.25). Therefore, the adsorption capacity of proteins to flavor compounds could be expressed as:

adsorption capacity (%)=100%−free flavor compound (%)

### 2.10. Statistical Analysis

The differences between the treatments were analyzed by one-way ANOVA (analysis of variance) under a Duncan’s multiple range test (*p* < 0.05). Pearson’s correlation analysis was conducted to show correlations between the different characters, using SPSS (Statistical Product and Service Solutions) software, version 19.0 (IBM Corporation, Chicago, IL, USA).

## 3. Results and Discussion

### 3.1. Proteolysis of MP by Commercial Proteases

**Figure 1:**
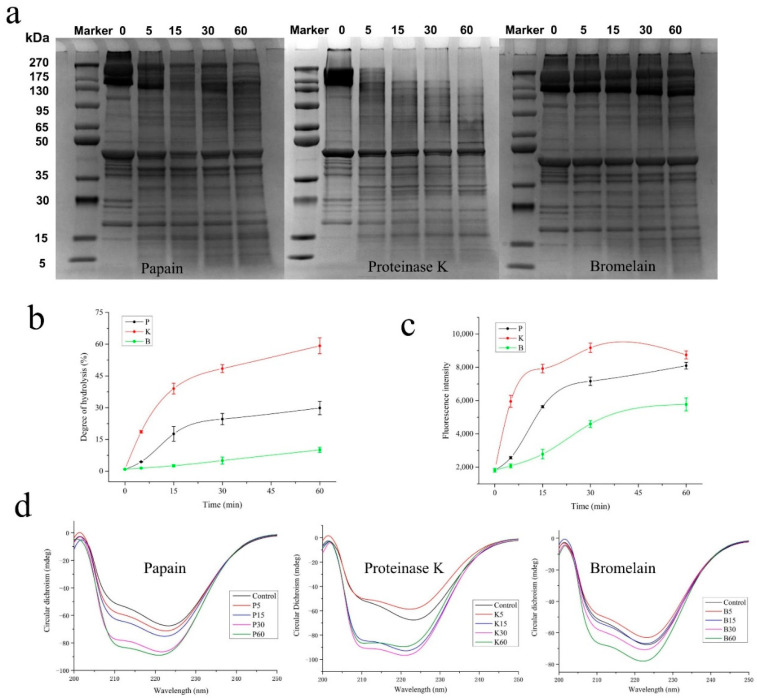
Effect of protease treatments on the changes in SDS-PAGE images (**a**) and DH (**b**), surface hydrophobicity (**c**) and CD spectra (**d**) of MP. DH and surface hydrophobicity measurements were repeated in triplicate.

The decomposition of MP during the protease treatment was analyzed by observing SDS-PAGE (Figure 1a) images and measuring the DH value (Figure 1b)**.** All the proteases were shown to hydrolyze MP, since the bands of the macromolecular proteins in MP hydrolyzed into smaller fractions gradually, the longer they were treated. Proteinase K exhibited the highest hydrolysis efficiency, which was followed by papain and bromelain. In addition, the enzymes presented stronger catalytic efficiency to hydrolyze larger molecules with a molecular weight higher than 200 kDa, which have been reported to consist of aggregation and myosin heavy chain [23]. By contrast, actin (43 kDa) was affected to a smaller degree, which disagreed with previous research [24] reporting that papain and bromelain showed a significant catalytic efficiency on actin. This may have been due to the use of enzymes from different brands, the diversity of isolation, and the purification method [25].

The DH value showed the released level of free α-NH_2_ from protein during the enzymatic treatment, reflecting the hydrolysis of the peptide bond. As shown in Figure 1b, the DH of protease-treated MP increased the longer they were treated. In addition, the DH increased rapidly in the first 15 min after the addition of the enzyme, and then increased slowly. These commercial proteases both contained exopeptidases and endopeptidases [26], resulting in the progressive degradation of the MP and an increase in the DH. In this section, proteinase K hydrolyzed the MP to the largest degree, which was followed by papain and bromelain.

### 3.2. Changes in Structures and Physicochemical Properties of MP Induced by Proteolysis

Hydrophobic interaction usually plays a key role in the interactions between protein and hydrophobic molecules. The surface hydrophobicity reflected the changes in the microenvironment of hydrophobic residues on the surface of the MP. As shown in Figure 1c, similar to the changes in the DH, the surface hydrophobicity of MP increased as the proteolytic treatment time increased, and the sample treated with proteinase K showed the most rapid increase in surface hydrophobicity. A previous study showed that trypsin treatment resulted in an increase in the surface hydrophobicity of myosin, depending on the dosage of the applied trypsin. In line with this study, the increase in the surface hydrophobicity of MP indicated the relationship between proteolysis and the increase in the hydrophobic residues of the treated protein [27]. These results could be attributed to the exposure of hydrophobic groups inside the protein, along with the hydrolysis of the protein. Notably, a slight decrease in surface hydrophobicity of MP was observed when the proteolysis treated by Pro K was 60 min. This finding could be explained by the collapse of the advanced structure of the protein after excessive hydrolysis, which then reduced the capacity of the treated MP and the ANS probes to combine.

The changes in the secondary structure of MP treated with proteases were analyzed by CD spectroscopy. As shown in Figure 1d, two negative peaks were found to locate at 208 nm and 222 nm. A downward shift in the peaks was observed in all the samples treated with a protease, which may have been due to the increase in protein solubility after enzymatic hydrolysis. In addition, the rate of the downward shift in the enzyme-treated groups was in line with the hydrolysis efficiency of the applied protease. Notably, the blue-shift of the spectra was also observed during the protease treatment, which suggested the transformation of the ordered structures into unordered structure [28]. The unfolding of the protein structure, caused by the enzymatic hydrolysis, may enhance the ability of the protein to bind to flavor compounds via exposure of sulfhydryl groups or other effective sites [29].

The values in the same column are significantly different (*p* < 0.05) when they are labeled by different lowercase letters (a–i). Each measurement was repeated in triplicate.

Protein solubility is defined as the proportion of protein dispersed in a buffer, or the corresponding level of dispersion, which has been an effective indicator to reflect the dispersion behavior of proteins [9]. As shown in Table 1, the salt solubility of the control was 61.13%. This value was increased to 79.99%, 76.88% and 81.86% in the samples treated with papain, proteinase K and bromelain, respectively, for 15 min. When the enzymatic treatment continued, the solubility of MP treated with papain decreased slightly, whereas the solubility of the bromelain-treated group continued to increase slightly. These results indicated that even a moderate enzymatic hydrolysis could improve the solubility of MP, which could be attributed to the reduction in molecular weight and the liberation of hydrophobic amino and carboxyl groups during proteolysis [30,31]. However, excessive proteolysis may lead to the exposure of hydrophobic groups that can interact, aggregate and cause a decrease in solubility [32]. Theoretically, an increase in solubility can elevate the amount of protein required to interact with a flavor compound, whereas many studies have shown the opposite results. Lv et al. (2017) also suggested that the solubility of myosin decreased, but its absorbing capacities to aldehydes and ketones increased along with an increase in the amount of added protease [26]. Shen et al. (2019) also revealed that the salt solubility of MP significantly decreased when the pH was decreased from 7.0 to 4.9, whereas its binding ability to 2,5-dimethylpyrazine reached its highest level at a pH of 5.5 [17]. Therefore, the relationship between protein solubility and its ability to bind flavor substances remains open to further exploration.

Turbidity has also been used as an indicator of protein dispersion behavior [33]. As the enzymatic hydrolysis process lengthened, the turbidity of the MP decreased significantly in all the groups, as compared to the control group (Table 1). This finding indicated that the degradation and the hydrolysis of the MP resulted in a better dispersion of hydrolysates. Furthermore, a relationship between the hydrolysis efficiency of the applied protease and the decreased rate of turbidity in the treated MP was found. For example, proteinase K showed the strongest hydrolysis efficiency, and the turbidity of the MP treated with proteinase K was halved (80.80 to 39.81 FTU) sharply, after only 5 min, and reached the lowest level after 15 min of treatment. The same results were reported, suggesting that changes in turbidity were highly related to the salt solubility of proteins [17].

The particle size was also analyzed to reflect the changes in dispersion behavior of the MP during hydrolysis (Table 1). It was shown that the particle size of the MP decreased gradually as the hydrolysis treatment lengthened. Again, proteinase K showed the highest efficiency in reducing particle sizes in the MP. This result was related to the degradation of the MP into smaller peptides and amino acids during proteolysis. In addition to the decreases in particle size, the specific surface area of the protein molecules increased, which may have improved the binding of the protein with other substances. A previous study indicated that the decrease in particle size improved the hydrophobic affinity of the protein nanoparticles to other substances [34]. Zhou et al. (2014) also reported that an increase in particle size induced by oxidation reduced the binding ability of MP with aldehydes and ketones [35].

### 3.3. Effect of Protease Treatments on the Quenching Ability of Flavor Compounds with MP

**Figure 2:**
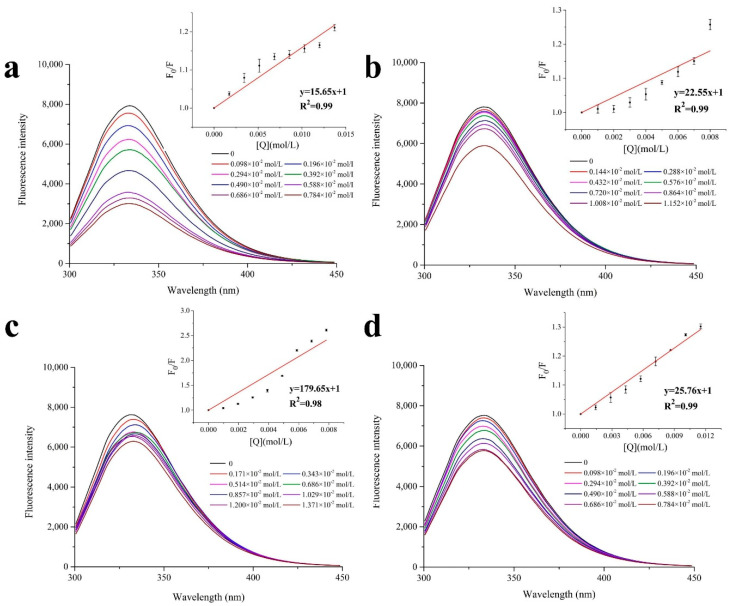
Fluorescence spectra analysis of the interaction between MP and butyraldehyde (**a**), 2-pentanone (**b**), octanal (**c**), and 2-octanone (**d**).

Fluorescence spectroscopy is a commonly used method to study the interactions between proteins and small molecules, since the intrinsic fluorescent intensity of aromatic residues, including phenylalanine (Phe), tyrosine (Tyr) and tryptophan (Trp) residues, are quenched when they are combined with small molecular weight collisions [36,37]. The fluorescence spectra of the untreated MP with a series of concentrations of the selected flavors are shown in Figure 2. In all groups, the peak of fluorescence emission spectra of the MP was approximately 340 nm. Along with the increase in the added flavor substance, the intensity of the emission decreased gradually, suggesting that all the selected aldehydes and ketones were effective quenchers of MP. The quenching of the MP induced by the selected flavors was evaluated by the K_sv_ values. The highest K_sv_ was found in octanal (K_sv octanal_ = 179.65), which was followed by K_sv 2-octanone_ = 25.76, K_sv 2-pentanone_ = 22.55, and K_sv butyraldehyde_ = 15.65. In both the aldehydes and the ketones, the longer the carbon chain, the larger the K_sv_ value obtained. Similar results were reported in a study by Shen et al. [20], which were attributed to the stronger hydrophobicity of ketones and aldehydes, along with the elongation of carbon chains. Notably, aldehydes had larger K_sv_ values than ketones when there are the same number of carbon chains, which was also reported in previous studies [38,39].

The fluorescence-quenching rate constant (K_q_) was calculated to further understand the interaction between the MP and the selected flavors, as affected by proteolysis (Table S1). All K_q_ values were larger than the maximum scattering collision quenching constant between biological macromolecules and quencher (2.0 × 10^10^ L·mol^−1^s^−1^), except for that of butyraldehyde. This result suggested that the fluorescence quenching of the MP by butyraldehyde was a static quenching, and both dynamic and static quenching occurred in the quenching of MP by other compounds [40]. The static quenching of butyraldehyde was due to its small steric hindrance effect that leads to its easier entry into the internal binding site of protein molecules [41]. For 2-pentanone, octanal and 2-octanone, a collision between the quencher and the MP also resulted in the fluorescence quenching of the MP, except for static quenching.

**Figure 3:**
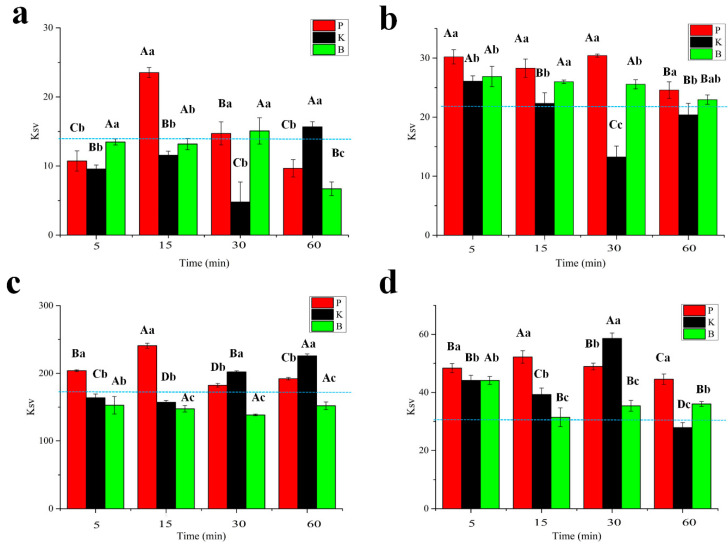
The K_sv_ of butyraldehyde (**a**), 2-pentanone (**b**), octanal (**c**), and 2-octanone (**d**) with MP treated by papain (P), proteinase K (K), and bromelain (B) for 0 (blue dotted line), 5, 15, 30, and 60 min. A–D indicates the significance level among the same samples treated for different times, while a–c indicates the significance level among the different enzyme treatments (*p* < 0.05). Each measurement was repeated in triplicate.

A comparison of the K_sv_ of the MP with the selected flavors is shown in Figure 3. Enzymatic treatment significantly changed the interaction between the MP and the flavor substances in a different way. As compared to the control sample (the dotted blue line), the enzymatic treatment generally increased the K_sv_ of the treated MP to ketones (Figure 3b,d) but decreased the K_sv_ of treated MP to aldehydes (Figure 3a,c), even though discrepancies were found among different groups depending on the applied protease and the treatment time. This may have been due to the difference in the polarity between aldehydes and ketones. Aldehydes have a higher polarity, better water solubility and relatively poor liposolubility compared to ketones, which are not conducive to hydrophobic binding to proteins [42].

**Figure 4:**
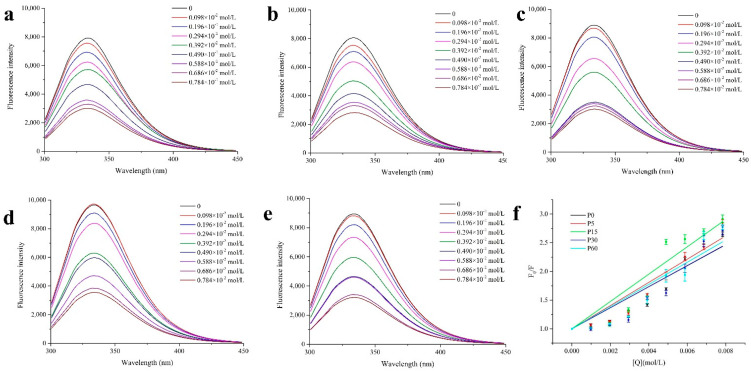
Fluorescence spectra analysis (**a**–**e**) and Stern–Volmer plots (**f**) of the interaction between octanal and the MP treated with papain at 0 (**a**), 5 (**b**), 15 (**c**), 30 (**d**) and 60 min (**e**).

Notably, the papain treated group generally showed the largest K_sv_ values among all the samples, especially in the sample treated within 15 min. By contrast, the bromelain treatment appeared to decrease the K_sv_ of the MP with butyraldehyde and octanal (Figure 3a,c, respectively) but increased the K_sv_ with 2-pentanone and 2-octanone (Figure 3b,d, respectively). In addition, along with the lengthening of the enzymatic treatment, the K_sv_ of the papain treated MP with butyraldehyde, octanal and 2-octanone increased in the initial 15 min and then decreased during the following procedure. This finding could indicate that a moderate treatment with papain enhanced the interaction between the MP and the flavor substances, while excessive processing impaired the ability of the MP to interact with these flavor compounds. Fluorescence spectra and Stern–Volmer plots of the samples treated with papain are shown in Figure 4, particularly to show the influence of the papain treatment on the interaction between octanal and the treated MP. In particular, the intensity of the intrinsic fluorescence of the MP treated with papain for 15 min weakened most rapidly as the octanal concentration increased (Figure 4c), which was in line with the largest slope of this sample in Figure 4f. Even though proteinase K has a higher hydrolysis efficiency than papain, proteinase K showed less influence on the interaction between the treated MP and the selected volatile compounds. This comparison indicated that the cleavage pattern with proteases may also play a crucial role in regulating the interplay between hydrolysates and volatile compounds.

### 3.4. Adsorption Ability of MP to Volatile Compounds Affected by Proteolysis and Correlation Analysis

**Figure 5:**
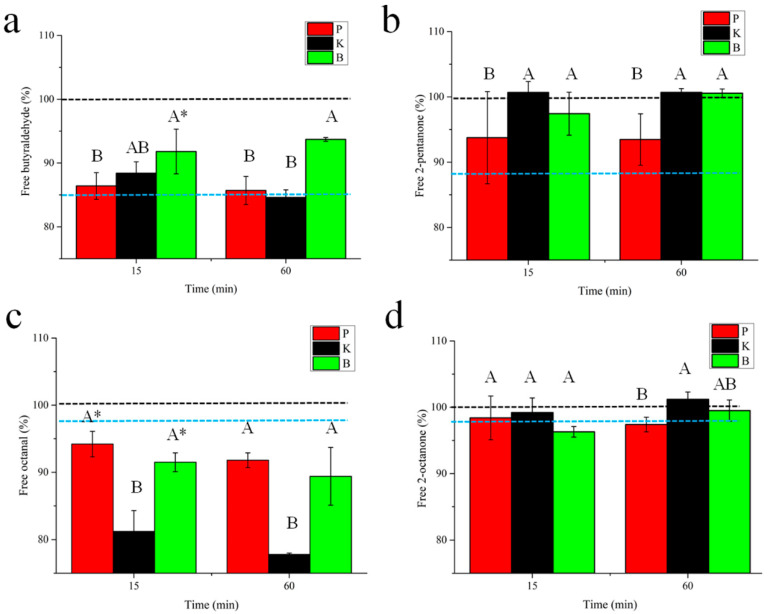
Changes in absorption of MP solution to butyraldehyde (**a**), 2-pentanone (**b**), octanal (**c**), and 2-octanone (**d**) affected by treatment with papain (P), proteinase K (K), or bromelain (B) for 0 (blue dotted line), 15, and 60 min. Uppercase letters A,B indicate significance level differences among different enzymes. Symbol * indicates significant differences between samples treated with the same enzyme for 15 and 60 min. Each measurement was repeated in triplicate.

The changes in the adsorption ability of the treated MP to selected volatile compounds, as induced by enzymatic treatment, were further explored. The levels of unabsorbed flavor by the controlled or hydrolyzed MP solution are compared in Figure 5. The untreated MP solution (blue dotted line) was shown to have the capacity to absorb all the selected flavors; proteolysis treatments were shown to affect the adsorption capacities of the MP. The adsorption capacity of the MP hydrolysate to octanal was increased, whereas the hydrolyzed MP showed reduced adsorption capacities to butyraldehyde and 2-pentanone. Notably, the type of enzyme, rather than the length of the enzyme treatment, appeared to have a greater influence on the adsorption capacity of the MP solution to the selected flavors. Bromelain treatments significantly reduced the adsorption capacity to butyraldehyde. Proteinase K or bromelain treatment largely reduced the adsorption capacity to 2-pentanone. The action of proteinase K elevated the adsorption capacity to 2-octanone to the largest degree, which was followed by bromelain and papain in descending order. In addition, the adsorption capacity of the MP to 2-octanone was only minorly affected by the proteolysis treatment.

**Figure 6:**
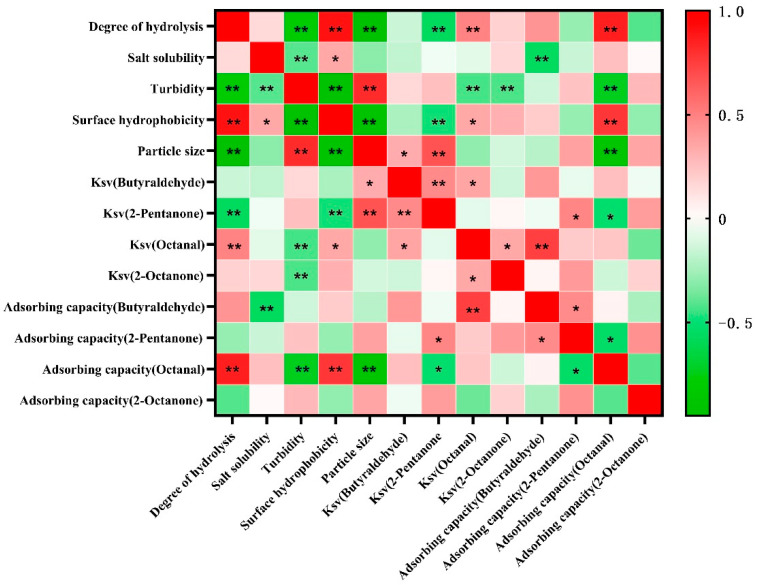
Heat map of the correlation analysis among degree of hydrolysis (DH) physicochemical properties, and K_sv_. The red color represents positive correlations and green indicates negative correlations. The significance is indicated as * *p* < 0.05; ** *p* < 0.01.

In the previous section, the type of applied protease and enzymatic treatment time were shown to affect the ability of the MP to interact with the selected flavor compounds. A correlation analysis was conducted to further explore the underlying mechanisms, and the results are shown in Figure 6. With the exception of salt solubility, the physicochemical properties including the DH, turbidity, surface hydrophobicity, and particle size all showed a significant relationship with each other. The relationship between the DH and the physicochemical properties indicated that protease treatment led to a reduction in protein size, better dispersion, and the exposure of hydrophobic groups in the MP. More importantly, a high correlation (r = 0.996) was found between the DH and surface hydrophobicity. Therefore, the surface hydrophobicity could be a crucial factor to be regulated and significantly affected the interaction between the protein hydrolysate and the selected flavor compounds. In addition, the DH value and surface hydrophobicity were both negatively related to the K_sv_ of 2-pentanone, whereas they were positively related to the K_sv_ adsorption capacity of octanal, possibly due to their different polarities. A high positive correlation (r = 0.859) was found between the adsorbing capacity of octanal and the DH. Proteolytic treatment could affect the gas–liquid interface surface tension of the MP solution, and the decrease in surface tension was reported to lead to a lower adsorbing capacity of the protein solution system [43]. There was a study reporting that the adsorbing capacity of MP to several kinds of ketones was consistent with the K_sv_, which was not found in this work [20].

An earlier study reported that the surface tension of MP was closely related to surface hydrophobicity, which influenced the adsorption behavior at the gas–liquid interface [44]. Guichard et al. also reported that hydrophobicity of proteins and flavor compounds showed a positive correlation during flavor–protein interactions [45]. The results in this study also suggested that an increase in the surface hydrophobicity may result in an increase in the K_sv_ value of papain treated MP that interacted with selected flavor substances within 15 min. However, the adsorption capacity of the protein solution should be affected in more complex ways, depending on the cleavage pattern of the applied protease. The papain treatment was shown to affect the interaction between the flavor compounds and the MP to the largest degree, whereas the adsorption capacity was more significantly affected by the proteinase K and bromelain treatment. Our previous study had indicated that the action of papain generated more free amino acids than bromelain and proteinase K, which could account for the different responses in K_sv_ and adsorption capacity to proteolysis treatment. Further studies are needed to explore this phenomenon.

## 4. Conclusions

Proteolysis was shown to change the interaction and adsorption capacity of an MP solution to butyraldehyde, 2-pentanone, octanal and 2-octanone. The surface hydrophobicity may be a crucial factor to be regulated, as it largely affected the interaction between the protein hydrolysate and the selected flavor compounds. The papain treatment was shown to affect the interaction between the flavor compounds and the MP to the largest degree, whereas the adsorption capacity was more significantly affected by the proteinase K and bromelain treatment. Additional research is needed to uncover the different responses of K_sv_ and adsorption capacity to proteolysis treatment.

## Figures and Tables

**Table 1:** Effect of different protease treatments on the changes in salt solubility, turbidity and particle size of MP.

Sample	Time (min)	Salt Solubility (%)	Turbidity (FTU)	Particle Size (10^3^ nm)
Control	-	61.13 ± 0.37 h	80.80 ± 1.96 a	15.43 ± 0.41 a
Papain	5	77.84 ± 1.78 de	57.79 ± 1.54 d	14.28 ± 0.43 b
	15	79.99 ± 2.73 bcd	40.53 ± 3.37 f	12.21 ± 0.51 cd
	30	75.70 ± 1.95 ef	36.25 ± 1.59 gh	10.39 ± 0.55 e
	60	74.14 ± 1.14 f	32.65 ± 1.67 hi	7.11 ± 0.48 g
Proteinase K	5	77.46 ± 1.79 def	39.81 ± 0.85 fg	9.26 ± 0.55 f
	15	76.88 ± 2.95 def	33.31 ± 1.08 hi	4.99 ± 0.44 h
	30	79.46 ± 1.54 cd	33.27 ± 1.52 hi	2.11 ± 0.09 i
	60	76.87 ± 1.88 def	31.39 ± 0.94 i	1.38 ± 0.04 i
Bromelain	5	65.73 ± 1.84 g	76.08 ± 3.50 b	14.98 ± 0.14 ab
	15	81.86 ± 2.60 bc	70.55 ± 4.03 c	12.78 ± 0.56 c
	30	82.88 ± 1.04 b	59.67 ± 0.74 d	11.63 ± 0.31 d
	60	89.12 ± 0.56 a	45.64 ± 2.32 e	9.58 ± 0.73 f

Values in the same column were significantly different (*p* < 0.05) when they are labeled by different lowercase letters (a–i). MP: myofibrillar proteins.

## Data Availability

Not applicable.

## Supplementary Materials

The following are available online at https://www.mdpi.com/article/10.3390/foods11060891/s1, Table S1: The K_sv_ and K_q_ values of flavor compounds in MP treated by enzymes at different time points.
