# Supplementary material for: Influence of Proteolysis on the Binding Capacity of Flavor Compounds to Myofibrillar Proteins
Source: Foods. 2022 Mar 21;11(6):891. doi: 10.3390/foods11060891 (PMC8955031; doi:10.3390/foods11060891)
Supplement: Supplementary file 1 [file foods-11-00891-s001.zip › foods-1581000-supplementary.pdf]

**Table S1.** The $K_{sv}$ and $K_q$ values of flavor compounds in myofibrillar proteinstreated by enzymes at different time points

| Enzyme | Flavor compounds | Time (min) | $K_{sv}$ ($L^{-1}mol$) | $K_q$ ($10^8$ $Lmol^{-1}s^{-1}$) | $R^2$ |
|---|---|---|---|---|---|
| Papain | Butyraldehyde | Control (0) | 14.30±1.36 | 14.30±1.36 | 0.9997 |
| | | 5 | 10.72±1.47 | 10.72±1.47 | 0.9999 |
| | | 15 | 23.52±0.73 | 23.52±0.73 | 0.9988 |
| | | 30 | 14.71±1.68 | 14.71±1.68 | 0.9973 |
| | | 50 | 9.67±1.25 | 9.67±1.25 | 0.9999 |
| | Octanal | Control (0) | 179.53±4.45 | 179.53±4.45 | 0.9850 |
| | | 5 | 203.78±1.29 | 203.78±1.29 | 0.9886 |
| | | 15 | 240.75±3.64 | 240.75±3.64 | 0.9850 |
| | | 30 | 182.19±2.70 | 182.19±2.70 | 0.9790 |
| | | 50 | 191.88±1.94 | 191.88±1.94 | 0.9805 |
| | 2-Pentanone | Control (0) | 22.57±2.37 | 22.57±2.37 | 0.9850 |
| | | 5 | 30.20±1.21 | 30.20±1.21 | 0.9997 |
| | | 15 | 28.28±1.54 | 28.28±1.54 | 0.9995 |
| | | 30 | 30.42±0.27 | 30.42±0.27 | 0.9999 |
| | | 50 | 24.58±1.39 | 24.58±1.39 | 0.9997 |
| | 2-Octanone | Control (0) | 25.17±0.65 | 25.17±0.65 | 0.9998 |
| | | 5 | 48.36±1.58 | 48.36±1.58 | 0.9997 |
| | | 15 | 52.22±2.17 | 52.22±2.17 | 0.9998 |
| | | 30 | 48.94±1.14 | 48.94±1.14 | 0.9983 |

| | | | | | |
|---|---|---|---|---|---|
| | | 50 | 44.55±1.78 | 44.55±1.78 | 0.9981 |
| | Butyraldehyde | Control (0) | 14.30±1.36 | 14.30±1.36 | 0.9997 |
| | | 5 | 9.55±0.60 | 9.55±0.60 | 0.9999 |
| | | 15 | 11.56±0.58 | 11.56±0.58 | 0.9999 |
| | | 30 | 4.79±2.88 | 4.79±2.88 | 0.9995 |
| | | 50 | 15.66±0.74 | 15.66±0.74 | 0.9996 |
| Bromelain | Octanal | Control (0) | 179.53±4.45 | 179.53±4.45 | 0.9850 |
| | | 5 | 163.73±5.53 | 163.73±5.53 | 0.9874 |
| | | 15 | 157.04±2.66 | 157.04±2.66 | 0.9781 |
| | | 30 | 201.90±1.71 | 201.90±1.71 | 0.9812 |
| | | 50 | 225.77±2.79 | 225.77±2.79 | 0.9778 |
| | 2-Pentanone | Control (0) | 22.57±2.37 | 22.57±2.37 | 0.9850 |
| | | 5 | 26.09±0.90 | 26.09±0.90 | 0.9998 |
| | | 15 | 22.33±1.79 | 22.33±1.79 | 0.9997 |
| | | 30 | 13.24±1.86 | 13.24±1.86 | 0.9999 |
| | | 50 | 20.38±1.95 | 20.38±1.95 | 0.9997 |
| | 2-Octanone | Control (0) | 25.17±0.65 | 25.17±0.65 | 0.9998 |
| | | 5 | 44.14±1.76 | 44.14±1.76 | 0.9997 |
| | | 15 | 39.30±2.21 | 39.30±2.21 | 0.9991 |
| | | 30 | 58.60±1.92 | 58.60±1.92 | 0.9993 |
| | | 50 | 27.88±1.70 | 27.88±1.70 | 0.9992 |
| Proteinase K | Butyraldehyde | Control (0) | 14.30±1.36 | 14.30±1.36 | 0.9997 |
| | | 5 | 13.47±0.44 | 13.47±0.44 | 0.9996 |
| | | 15 | 13.18±0.81 | 13.18±0.81 | 0.9999 |
| | | 30 | 15.07±1.90 | 15.07±1.90 | 0.9997 |

| | | | | |
|---|---|---|---|---|
| | 50 | 6.70±0.98 | 6.70±0.98 | 0.9999 |
| Octanal | Control (0) | 179.53±4.45 | 179.53±4.45 | 0.9850 |
| | 5 | 152.75±12.90 | 152.75±12.90 | 0.9850 |
| | 15 | 147.43±4.76 | 147.43±4.76 | 0.9833 |
| | 30 | 138.38±1.34 | 138.38±1.34 | 0.9896 |
| | 50 | 151.94±5.71 | 151.94±5.71 | 0.9873 |
| 2-Pentanone | Control (0) | 22.57±2.37 | 22.57±2.37 | 0.9850 |
| | 5 | 26.87±1.72 | 26.87±1.72 | 0.9999 |
| | 15 | 25.99±0.30 | 25.99±0.30 | 0.9998 |
| | 30 | 25.56±0.77 | 25.56±0.77 | 0.9998 |
| | 50 | 22.96±0.78 | 22.96±0.78 | 0.9999 |
| 2-Octanone | Control (0) | 25.17±0.65 | 25.17±0.65 | 0.9998 |
| | 5 | 44.12±1.37 | 44.12±1.37 | 0.9995 |
| | 15 | 31.44±3.24 | 31.44±3.24 | 0.9999 |
| | 30 | 35.38±1.86 | 35.38±1.86 | 0.9985 |
| | 50 | 36.02±0.83 | 36.02±0.83 | 0.9997 |
